# Heparanase Promotes Engraftment and Prevents Graft versus Host Disease in Stem Cell Transplantation

**DOI:** 10.1371/journal.pone.0010135

**Published:** 2010-04-15

**Authors:** Menachem Bitan, Lola Weiss, Michael Zeira, Eyal Zcharia, Shimon Slavin, Arnon Nagler, Israel Vlodavsky

**Affiliations:** 1 Department of Bone Marrow Transplantation, Hadassah-Hebrew University Medical Center, Jerusalem, Israel; 2 Department of Oncology, Hadassah-Hebrew University Medical Center, Jerusalem, Israel; 3 The Division of Hematology and Bone Marrow Transplantation, Chaim Sheba Medical Center, Tel-Hashomer, Israel; 4 Cancer and Vascular Biology Research Center, The Bruce Rappaport Faculty of Medicine, Technion, Haifa, Israel; New York University, United States of America

## Abstract

**Background:**

Heparanase, endoglycosidase that cleaves heparan sulfate side chains of heparan sulfate proteoglycans, plays important roles in cancer metastasis, angiogenesis and inflammation.

**Design and Methods:**

Applying a mouse model of bone marrow transplantation and transgenic mice over-expressing heparanase, we evaluated the effect of heparanase on the engraftment process and the development of graft-versus-host disease.

**Results:**

Analysis of F1 mice undergoing allogeneic bone marrow transplantation from C57BL/6 mice demonstrated a better and faster engraftment in mice receiving cells from donors that were pretreated with heparanase. Moreover, heparanase treated recipient F1 mice showed only a mild appearance of graft-versus-host disease and died 27 days post transplantation while control mice rapidly developed signs of graft-versus-host disease (i.e., weight loss, hair loss, diarrhea) and died after 12 days, indicating a protective effect of heparanase against graft-versus-host disease. Similarly, we applied transgenic mice over-expressing heparanase in most tissues as the recipients of BMT from C57BL/6 mice. Monitoring clinical parameters of graft-versus-host disease, the transgenic mice showed 100% survival on day 40 post transplantation, compared to only 50% survival on day 14, in the control group. *In vitro* and *in vivo* studies revealed that heparanase inhibited T cell function and activation through modulation of their cytokine repertoire, indicated by a marked increase in the levels of Interleukin-4, Interleukin-6 and Interleukin-10, and a parallel decrease in Interleukin-12, tumor necrosis factor-alfa and interferon-gamma. Using point mutated inactive enzyme, we found that the shift in cytokine profile was independent of heparanase enzymatic activity.

**Conclusions:**

Our results indicate a significant role of heparanase in bone marrow transplantation biology, facilitating engraftment and suppressing graft-versus-host disease, apparently through an effect on T cell activation and cytokine production pattern.

## Introduction

Heparan sulfate proteoglycans (HSPGs) are ubiquitous macromolecules associated with the cell surface and extracellular matrix (ECM) of a wide range of cells [1], [2], [3]. The basic HSPG structure consists of a protein core to which several linear heparan sulfate (HS) chains are covalently O-linked [1], [2], [3]. HS chains, unique in their ability to bind a multitude of proteins, ensure that a wide variety of bioactive molecules bind to the cell surface and ECM and thereby function in the control of diverse normal and pathological processes [1], [4], [5]. The majority of studies on cell interaction with the microenvironment focused, among other approaches, on proteolytic enzymes [6]. The involvement of glycosaminoglycan (e.g., heparan sulfate) degrading enzymes (e.g., heparanase) was underestimated, primarily due to a lack of appropriate molecular probes to explore their causative role in cell-ECM interactions and related effects. A long-term research on the biology of heparanase led to the cloning of a single gene encoding a HS-degrading endoglycosidase (heparanase) which plays important roles in cancer metastasis, angiogenesis and inflammation [7], [8], [9], [10], [11], [12], [13], [14], [15], [16], [17]. Heparanase is synthesized as a 65 kDa latent precursor that subsequently undergoes proteolytic processing by cathepsin L [18], [19], yielding 8 kDa and 50 kDa protein subunits that undergo heterodimerization to form the active enzyme [20], [21], [22]. The enzyme has been identified in invasive normal and malignant cells, including activated cells of the immune system, cytotrophoblasts, keratinocytes, lymphoma, melanoma, myeloma and carcinoma cells [7], [8], [9], [10], [11], [12], [13], [14].

Extravasation of circulating hematopoietic and immune cells is accompanied by degradation of various components of the subendothelial ECM. Activated immune cells produce and secrete a variety of ECM degrading enzymes, including heparanase [10], [11], [23], [24], [25]. Degradation of HS disintegrates the supramolecular structure of the subendothelial basal lamina, consequently facilitating trans-endothelial migration of neutrophils and activated lymphocytes, thereby mediating their extravasation during immune responses [10], [11], [15], [23], [24], [25]. Allogeneic hematopoietic stem cell transplantation (SCT) is a therapeutic modality in a growing number of malignant and non-malignant diseases. It provides a powerful anti-tumor activity through the graft-versus-leukemia/tumor effect mediated by donor T cells [26], [27], [28]. Since donor alloreactive T-cells are also being activated against host epitopes presented on normal tissues, graft-versus-host disease (GVHD) [29], [30] is the most common threatening complication post allogeneic transplantation.

We have recently demonstrated that heparanase modulates the bone marrow (BM) microenvironment as well as basic features of hematopoietic stem and progenitor cells, including development, proliferation and retention [31]. We have also found a marked increase in the number of hematopoietic stem cells in the BM of heparanase over-expressing transgenic (*Hpa-tg*) mice compared to wild type (*wt*) control mice [31]. Moreover, a minimal dose of white blood cells from the BM of *hpa-tg* mice, but not *wt* mice, was sufficient to rescue lethality irradiated C57BL/6 recipient mice, indicating that a higher number of hematopoietic repopulating cells exists in the BM of the *hpa-tg* mice [31]. These results and the recently reported protective effect of heparanase against autoimmune type-1 diabetes [32] prompted us to investigate the effect of heparanase on engraftment and GVHD in transplantation animal models.

In the GVHD process, antigen-specific CD4+ cells polarized toward the Th1 phenotype mediate inflammatory damage in the host body, resulting in tissue dysfunction, multi-organ failure and high mortality rate [26], [28], [29], [30]. On the other hand, increased production of Th2 cytokines and a parallel suppression of Th1 cytokines, lead to amelioration of GVHD [28], [33], [34], [35]. Mature T cells infused with the bone marrow graft respond to alloantigens and other changes in the allogeneic host tissues induced by the pre-transplantaion conditioning. The injured host tissues produce proinflammatory cytokines such as interleukin-2 (IL-2) and interferon-gamma (IFN-γ) which in turn activate donor effector cells to release IL-1 and tumor necrosis factor-alfa (TNF-α), further activating the alloreactive T cells, thereby causing direct tissue destruction [36], [37], [38], [39].

Our results indicate an enhancing effect of heparanase on engraftment, as well as a protective activity against GVHD. The anti-GVHD effect appears to be attributed to suppression of T cell activation and a shift from a Th1 to Th2 cytokine profile.

## Materials and Methods

### Mice

Eight to 12 weeks old C57BL/6 (H-2^b^) and (Balb/c x C57BL/6) (H-2^d/b^) F1 mice, male and female, were purchased from Harlan Breeding Facility (Jerusalem, Israel). Heparanase overexpressing C57BL/6 transgenic (*hpa*-*tg*) mice were previously described in detail [40]. The heparanase transgene was introduced under the actin promoter to drive overexpression of heparanase in most tissues [40]. All mice were maintained in top filtered cages in a standard animal facility. Cages, sawdust and water bottles were autoclaved weekly. All the animal experiments were approved by the animal committees of the Hebrew University, Jerusalem Israel (MD-89.49-4) according to the NIH guidelines

### Conditioning by radiation

F1 and *Hpa-tg* mice were exposed to a sublethal (750 cGy) total body irradiation by a linear accelerator (Varian Clinac 6X GC, Palo Alto, CA) at a dose rate of 170 cGy/min, and a source-to-skin distance of 80 cm [31].

### Spleen cell preparation

Spleen cells from donor mice were suspended in PRMI 1640 medium supplemented with 10% fetal calf serum, glutamine (2 mM), penicillin (100 U/ml) and streptomycin (100 µg/ml) (Biological Industries, Beit-Haemek, Israel), washed twice and resuspended in the same medium [41].

### Transplantation design

One day following irradiation, F1 mice were transplanted intravenously with 10×10^6^ allogeneic (C57BL/6) spleen cells. Animals were divided into 4 groups (10 mice each): mice transplanted with spleen cells obtained from C57BL/6 mice pretreated with saline (3–5 days) followed by daily intraperitoneal (i.p) injection of saline (group 1), or recombinant heparanase (group 2), post transplantation; mice transplanted with spleen cells obtained from C57BL/6 mice pretreated with heparanase (3–5 days, i.p.) followed by daily injection of saline (group 3), or recombinant heparanase (group 4) for 7 days post transplantation. The first injection was given 15 min before transplantation. In another set of experiments, *hpa-tg* and *wt* mice were injected with 25×10^6^ or 50×10^6^ C57BL/6 splenocyes one day post irradiation. One month after transplantation, the number of donor-type cells present in the blood was determined by flow cytometry. Chimerism was assessed by the ameloginin gene expression method, as previously described [42].

### Monitoring of GVHD

Recipient mice were monitored for clinical signs of GVHD (ruffled fur, diarrhea, runt disease, weight loss) and survival, as described [41].

### Heparanase

Single-chain GS3 active heparanase gene construct, composed of the 8 kDa and 50 kDa heparanase subunits, was kindly provided by Dr. Christian Steinkuhler (IRMB/Merck Research Laboratories, Pomezia, Italy) [22], and the protein was purified from the conditioned medium of baculovirus infected cells, as described [21], [22]. Recombinant 65 kDa latent human heparanase and inactive heparanase mutated in glutamic acid residues 225 and 343 that comprise the enzyme active site [43] were purified from the culture medium of transfected HEK-293 cells, as described [44]. Heparanase preparations were assayed for the presence of bacterial endotoxin by Biological Industries (Beit Haemek, Israel), using the gel-clot technique (Limulus amebocyte lysate–LAL test) and were found to contain <10 pg/ml endotoxin.

### Modified heparin

Compound ST1514 was kindly provided by Dr. Claudio Pisano (Sigma-Tau, Research Department, Pomezia, Italy). Briefly, heparin was subjected to controlled alkali-catalyzed removal of sulfate groups of iduronic acid 2-O-sulfate residues, giving rise to the corresponding epoxide derivative. The epoxide rings were opened, followed by oxidative glycol-splitting of the newly formed (and the preexisting) nonsulfated uronic acid residues [45], [46]. The ST1514 compound is 50% glycol-split modified heparin (H^50^gs; MW 11,200) [45], [46].

### T-cell activation

#### a) ConA

Mouse spleen cells were cultured in RPMI-1640 medium supplemented with 10% heat-inactivated human AB serum in flat-bottomed 96-well microtiter plates (Nunc, Wiesbaden, Germany) containing 0.5×10^6^ cells/well/0.2 ml. Response to 2 µg/ml concanvalin-A (ConA, Sigma, St. Louis, MO) was assessed by ^3^H-thymidine incorporation, as described [47].

#### b) Mixed lymphocyte culture (MLC)

Mouse spleen cells were cultured in RPMI-1640 medium, supplemented with 10% heat-inactivated human AB serum in 96-well flat-bottomed microtiter plates (Nunc, Wiesbaden, Germany) containing 1×10^6^ responding cells and 1×10^6^ irradiated (3000 cGy) stimulating cells per 0.2 ml well. Cells were cultured for 5 days in a 5% CO_2_ humidified incubator. Twenty hours before harvesting, 1 µCi ^3^H-thymidine was added to each well and thymidine incorporation was measured, as described [47].

### Cytotoxicity assay

ConA activated splenocytes were co-cultured with 3–5×10^6^ target Yac (H-2^a^, NK-sensitive tumor cell line) cells [48], with or without active (5 µg/ml) or latent (30 µg/ml) heparanase in order to evaluate their killing capacity. The Yac cells were first incubated overnight with 5 µl ^35^S-methionine (Easytag methionine, L-[^35^S], TBq/mmol; Perkin Elmer Life Science, Boston, MA) in RPMI medium without methionine. Cytotoxic activity was measured in a 5 h ^35^S-release assay, as previously described [48].

### Cytokine analysis

In order to assess Th1 vs. Th2 cell phenotypes, medium from C57BL/6-derived spleen lymphocytes, cultured for 24 h in the absence or presence of heparanase, was subjected to ELISA analysis of IL-4, IL-6, IL-10, IL-12, IFN-γ and TNF-α, as described [38], [39], [41], [49].

### Statistics

Student's *t* test was used for statistical analysis of the results. Asterisk (*) indicates statistical difference (p<0.05). Two Asterisks (**) indicate highly statistical differences (p<0.01).

## Results

### Effect of heparanase on engraftment

**Figure 1:**
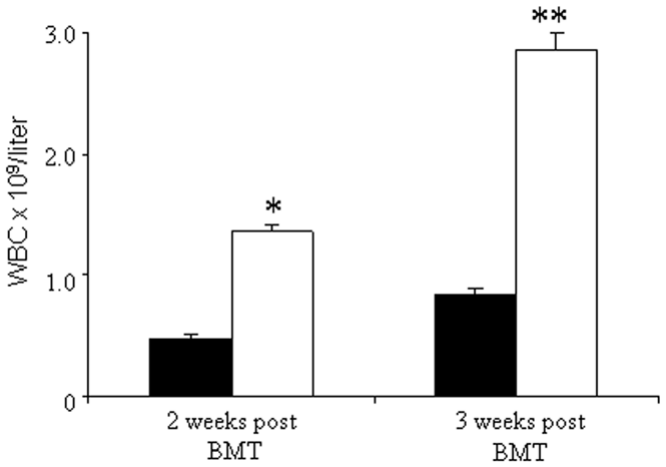
Heparanase potentiates engraftment of WBC. F1 mice were sublethally irradiated (750 cGy) and transplanted intravenously with 10×10^6^ spleen cells taken from heparanase (5 µg/mouse/day, i.p. for 5 days) or saline (control) treated C57BL/6 mice. The recipient mice were treated with heparanase (5 µg/mouse/day, i.p.) from the day of transplantation until day +7. Control recipient mice were injected with saline alone. Each group consisted of 8 mice. Heparanase treatment of both the donor and recipient mice caused a significant increase in the mean WBC count on day +14 post transplantation. 1.36×10^9^/L (range 1.2–1.68×10^9^/L) (□) vs. 0.48×10^9^/L (range 0.3–0.74×10^9^/L) in the control group (▪). Significantly higher WBC counts were maintained in the heparanase treated group 3 weeks post transplantation. Chimerism was assessed by the ameloginin gene expression method. Each bar represents mean ± SD (n = 8 mice) and the experiment was performed 3 times with similar results.

Heparanase facilitates release of ECM-bound growth factors and chemokines via degradation of heparan sulfate [9], [16]. Hence, we investigated whether heparanase enhances engraftment of hematopoietic cells. F1 mice were sublethally irradiated (750 cGy) and transplanted with 10×10^6^ spleen cells derived from heparanase (5 µg/mouse/day, i.p. for 3 days)- or saline (control)- treated C57BL/6 mice. The recipients were injected with recombinant heparanase (5 µg/mouse/day, i.p.) from the day of transplantation until day +7. Control recipient mice were injected with saline alone. Heparanase treatment of both the donor and recipient mice resulted in a significant shortening of time to engraftment. The mean WBC counts on day +14 post transplantation in the heparanase treated mice was 1.36×10^9^/L (range 1.2–1.68×10^9^/L), as compared to 0.48×10^9^ cells/L (range 0.3–0.74×10^9^/L) in the control group (p<0.01). Significantly higher counts were obtained after 3 weeks, 2.85×10^9^ cells/L (range 2.4–3.4×10^9^/L) vs. 0.85×10^9^ cells/L (range 0.6–1.2×10^9^/L), respectively (Fig. 1) (p<0.001). Donor engraftment was confirmed by the ameloginin gene expression method [42] (not shown).

### Effect of heparanase on GVHD

#### Prolonged survival of mice treated with heparanase

**Figure 2:**
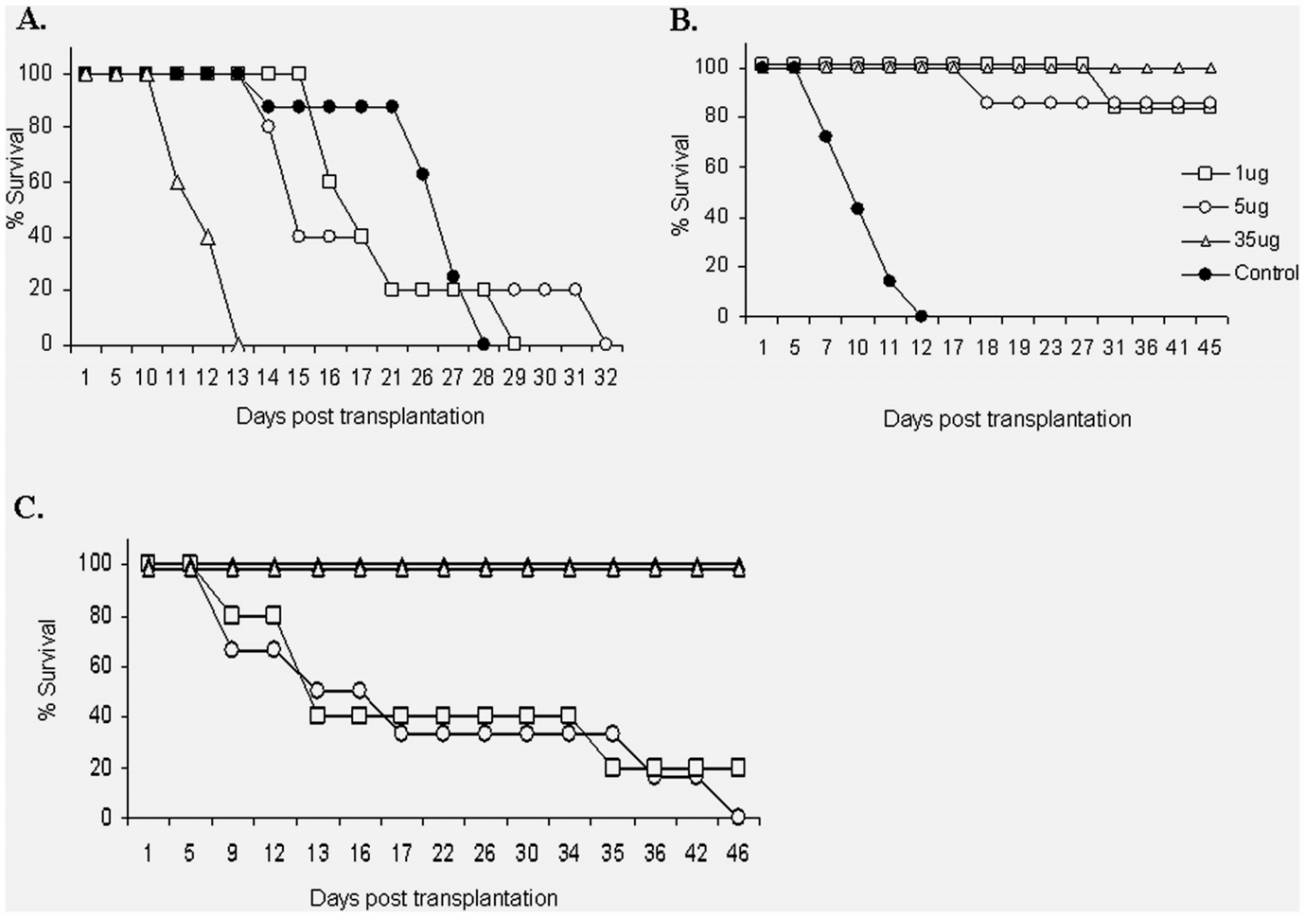
Effect of heparanase on GVHD. **A. Prolonged survival of mice treated with heparanase.** Mice were sublethally irradiated (750 cGy) and transplanted i.v with 10×10^6^ spleen cells from heparanase treated or un-treated C57BL/6 mice (5 µg/mouse/day, i.p. for 3 days). The recipient mice were injected with heparanase (5 µg/mouse/day, i.p. daily) from the day of transplantation until day +7, or with saline for the same period of time. Altogether, 4 experimental groups (10 mice each) were tested: Donor and recipient mice treated with heparanase (•); Only donor mice treated with heparanase (□); Only recipient mice treated with heparanase (○); Donor and recipient mice treated with saline alone (Δ). A significant prolongation of survival was documented when both the donor and recipient mice were treated with heparanase (•), as compared to the control group where both the donor and recipient mice were treated with saline alone (Δ). In the two other groups, where heparanase was administered to either the donors (•) or recipients (□), a partial effect was achieved. **B. Mice treated with different doses of heparanase.** Mice were sublethally irradiated (750 cGy) and transplanted with 10×10^6^ spleen cells from heparanase treated C57BL/6 mice (5 µg/mouse/day, i.p. for 3 days). The recipient mice were sub-grouped with each arm (8 mice each) receiving a different dose of heparanase per day. Injections were given from the day of transplantation as follows: 1 µg/mouse/day, for 7 days until day +7 post transplantation (□); 5 µg/mouse/day, for 7 days until day +7 post transplantation (○); 35 µg/mouse twice weekly (Δ); Both the donor and recipient mice treated with saline alone, as control (•). All the control mice died of GVHD. In contrast, all the mice treated with 35 µg heparanase/mouse and all the mice (except one in each group) in the two other groups, remained alive until the end of the experiment (>45 days). Mice that received 1 µg heparanase/mouse/day exhibited clinical signs of mild GVHD. **C. Transgenic mice over-expressing heparanase.** Spleen-derived progenitor cells obtained from C57BL/6 mice were injected with 25×10^6^ cells/mouse (▵), or 50×10^6^ cells/mouse (▴) into heparanase transgenic (*Hpa-tg*) and control mice (n = 10). All the *Hpa-tg* mice survived until the end of the experiment. In contrast, more than 80% of the control mice, receiving either 25×10^6^ cells/mouse (□), or 50×10^6^ cells/mouse (○), died of GVHD. The experiment was performed twice with similar results.

F1 mice were sublethally irradiated (750 cGy) and transplanted with 10×10^6^ spleen cells from heparanase treated or untreated C57BL/6 mice (5 µg/mouse/day, i.p. for 3 days). The recipient mice were injected with heparanase (5 µg/mouse/day, i.p. daily) or saline from the day of transplantation until day +7. Thus, the 4 experimental groups were as follows: a) Both donor and recipient mice treated with heparanase; b) Only donor mice treated with heparanase; c) Only recipient mice treated with heparanase; d) Both donor and recipient mice treated with saline. Figure 2A demonstrates a highly significant prolongation in survival when both the donor and recipient mice were treated with heparanase (group a) as compared to group (d) where both the donor and recipient mice received saline alone. A partial effect was achieved when either the donor or recipient mice received heparanase. Next, F1 recipient mice were sub-grouped and received different doses of heparanase (1 µg/mouse/day, 5 µg/mouse/day) for 7 days starting on the day of transplantation, or 35 µg heparanase per mouse, twice weekly for 5 weeks. Control mice received saline instead of heparanase. While all mice in the control group died of GVHD, all mice treated with 35 µg heparanase twice weekly and the vast majority of mice receiving 1 and 5 µg heparanase daily, remained alive for >45 days. Mice receiving 1 µg heparanase displayed mild signs of GVHD (Fig. 2B).

#### Prolonged survival of transgenic mice over-expressing heparanase

We investigated the outcome of BMT in transgenic (*hpa-tg*) mice over-expressing the heparanase gene in most tissues [40]. For this purpose, *hpa-tg* and control host mice were injected with 10×10^6^ spleen cells obtained from C57BL/6 mice. Mice were evaluated for the extent of GVHD and survival time. The clinical parameters of follow-up included weight loss, hair loss and diarrhea. All the donor mice were male and the recipient mice were females and engraftment was monitored by ameloginin gene expression [42] (not shown). As demonstrated in figure 2C, the *hpa-tg* mice failed to develop clinical signs of GVHD and all mice survived at least 45 days post transplantation, when the experiment was terminated. On the other hand, more than 80% of the control mice developed GVHD and died during the same period of time (Fig. 2C). Similar results were obtained when the number of injected donor cells was increased to 25×10^6^, or even 50×10^6^ cells/mouse, further supporting the protective effect of heparanase generated by the *hpa-tg* mice (Fig. 2C).

### Heparanase regulates T cell function

#### Reduced activation and killing capacity of splenocytes in vitro

**Figure 3:**
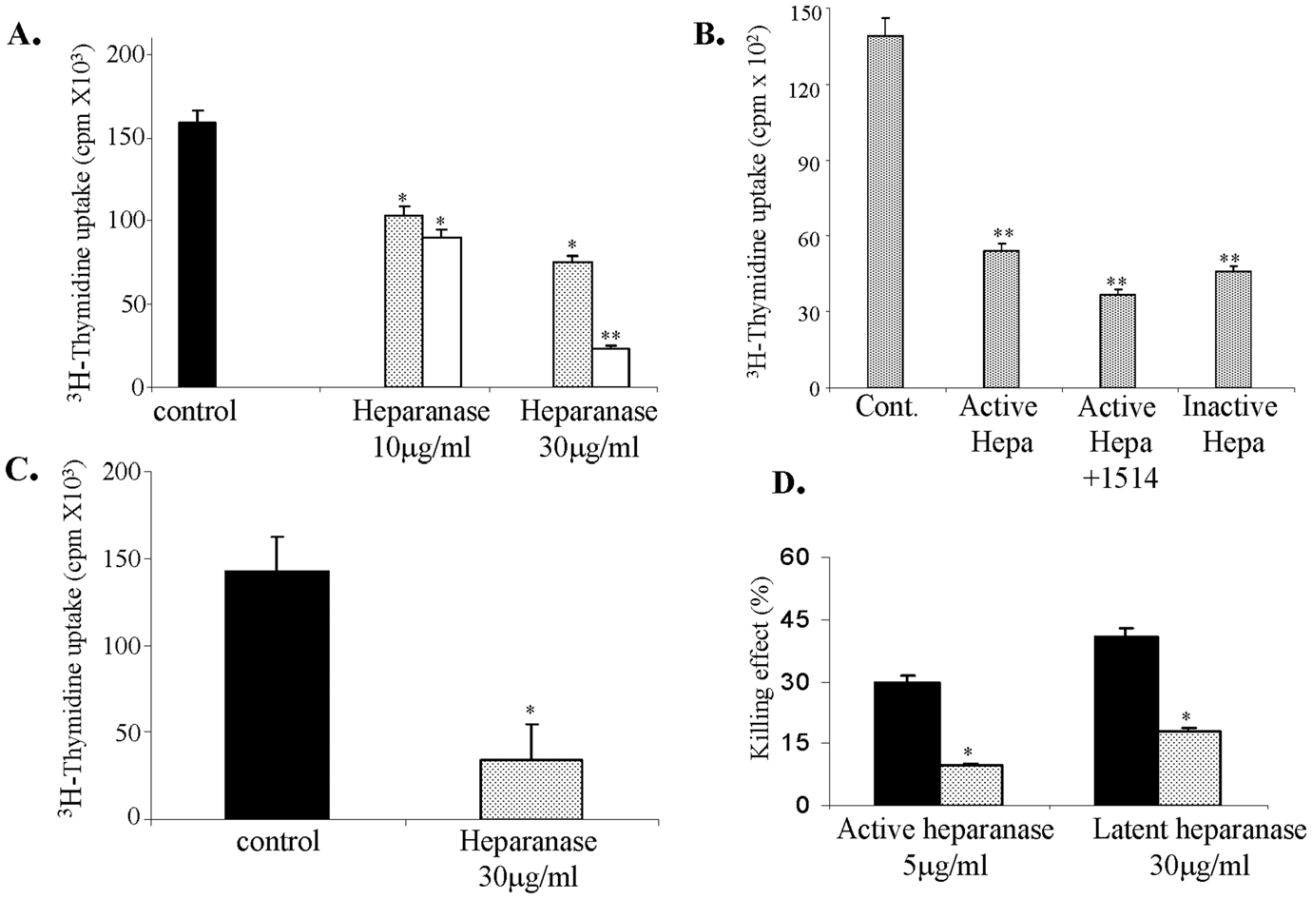
Effect of heparanase on activation of T lymphocytes. **A. ConA activation.** Mouse spleen cells were isolated and subjected to activation with ConA in the absence (control) (▪) and presence of 10 or 30 µg/ml recombinant latent (65 kDa) (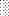) or active (8+50 kDa) (□) heparanase, followed by measurements of ^3^H-thymidine incorporation, as described under ‘Materials & Methods’. Addition of heparanase to the culture medium resulted in a significant (p<0.01) dose dependent decrease in ConA activation and proliferation of the spleen cells. The asterisk (*) indicates statistically significant differences between the control and the different treatments. **B. Heparanase-mediated inhibition of ConA stimulated T-cell proliferation is independent of its enzymatic activity.** Mouse spleen cells were isolated and subjected to activation with ConA in the absence (control) and presence of active heparanase, active heparanase plus glycol split heparin (100 µg/ml, compound 1514), or inactive heparanase (point mutated in glutamic residues 225 and 343). ^3^H-thymidine incorporation was inhibited to a similar extent regardless of whether the heparanase was enzymatically active or inactive (p<0.001). **C. Mixed lymphocyte culture (MLC).** One way MLC reaction was performed in the absence (control) (▪) or presence (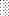) of 30 µg/ml recombinant latent (65 kDa) heparanase. A marked decrease in activation (^3^H-thymidine incorporation) of Balb/c-derived lymphocytes sensitized against C57BL/6-derived lymphocytes was noted in the heparanase treated culture (p<0.01). **D. Killing capacity of activated T cells.** ConA activated splenocytes were co-cultured with target Yac cells in the absence (▪) or presence (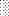) of 5 µg/ml active (8+50 kDa) or 30 µg/ml latent (65 kDa) heparanase in order to evaluate their killing capacity. Treatment with either the latent or active forms of heparanase markedly inhibited the ability of the activated lymphocytes to kill their target cells (p<0.01). Each bar represents mean ± SD from triplicate wells. All experiments were performed at least three times; variations between different experiments did not exceed ±20%.

GVHD is characterized by an attack of alloimmune donor T-cells on host tissues and organs. CD4^+^ cells are polarized toward the Th1 phenotype and thereby mediate the inflammatory process through which tissue damage occurs [28], [33], [34], [35], [50]. Having demonstrated a protective effect of heparanase against GVHD in mice, and in an attempt to elucidate the mode of heparanase action in this process, we investigated its effect on activation of lymphocytes. For this purpose, mouse splenocytes were cultured (12 h, 37°C) without or with increasing amounts of either active (8+50 kDa) or latent (65 kDa) heparanase, and exposed to ConA to induce cell proliferation. Addition of heparanase to the culture medium resulted in a substantial, dose dependent decrease in ConA activation and proliferation of the spleen cells (Fig. 3A). The Active (8+50 kDa) form of heparanase was more potent than the latent 65 kDa pro-enzyme (Fig. 3A). A similar decrease in ConA activation was obtained in the absence or presence of 100 µg/ml of the heparanase inhibitor ST1514 (glycol-split heparin [15], [45], [46]) (Fig. 3B). Under this condition, heparanase activity was fully inhibited, further emphasizing that heparanase enzymatic activity is not required for its inhibition of lymphocyte activation. In order to verify this finding, we utilized heparanase in which glutamic acid residues 225 and 343 that comprise the enzyme active site [43] were point mutated, yielding an inactive enzyme, as previously described [51]. The inactive enzyme inhibited the activation of lymphocytes by ConA to an extent comparable in magnitude to that of active (8 + 50 kDa) heparanase (Fig. 3B), further substantiating that a non-enzymatic activity of heparanase is responsible for its inhibition of ConA induced lymphocytes activation. In a subsequent experiment, spleen cells were taken from Balb/C mice and reacted against C57BL/6 splenocytes in a one way mixed lymphocyte culture (MLC), in the absence or presence of 30 µg/ml latent heparanase. As demonstrated in figure 3C, latent heparanase markedly inhibited (∼4 fold) the MLC response. Similar results were obtained using 5 µg/ml of the active enzyme (not shown).

#### Killing capacity of activated splenocytes

Spleen derived lymphocytes were subjected to ConA activation and the activated lymphocytes were then co-cultured with target Yac cells, with or without addition of active (5 µg/ml) or latent (30 µg/ml) heparanase, in order to evaluate their killing capacity. Incubation with either the active or latent enzyme, caused a marked reduction (3- and 2-fold, respectively) in the capacity of the ConA activated lymphocytes to kill their target cells. Thus, heparanase appears to suppress not only the activation of lymphocytes, but also their killing capacity (Fig. 3D), resulting in a robust lymphosuppressive effect.

#### Effect of heparanase on cytokine production in vivo

**Figure 4:**
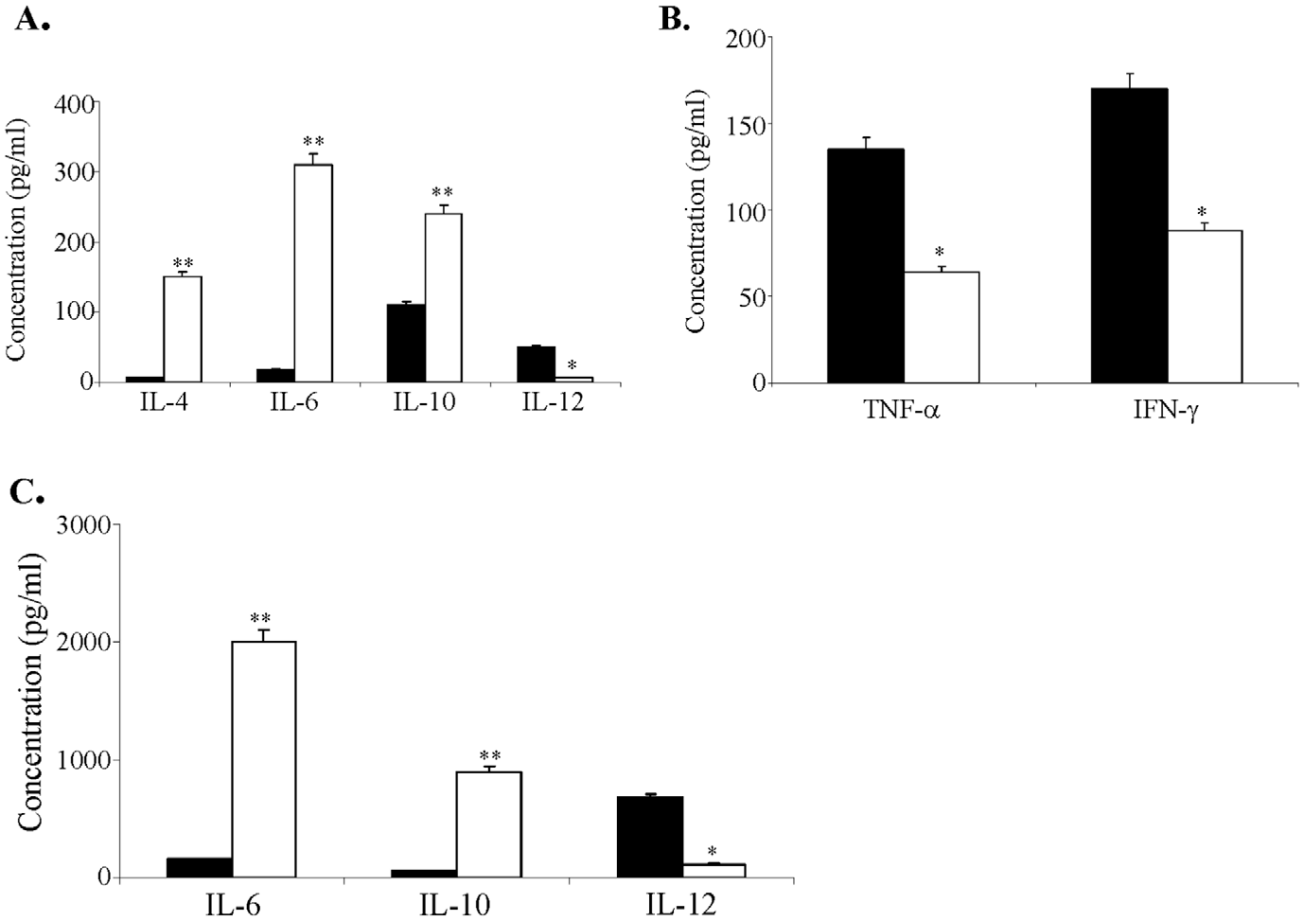
Effect of heparanase on cytokine production. **A, B. In vivo. A.** C57BL/6 mice were subjected to a daily injection of active (8+50 kDa) heparanase (3 days, 5 µg/mouse/day) or saline (control). Splenocytes were then harvested, activated with ConA (24 h, 37°C, RPMI + 10% FCS) and aliquots of the culture medium were subjected to ELISA analysis of IL-4, IL-6, IL-10 and IL-12. The amounts of secreted Th2-type cytokines such as IL-4, IL-6 and IL-10, were increased following *in vivo* administration of heparanase (□) vs. saline (▪). In contrast, under the same conditions, there was a marked decrease in the level of IL-12, representing a Th1-associated cytokine. **B. TNF-α and IFN-γ.** C57BL/6 mice were subjected to a daily injection of active (8+50 kDa) heparanase (3 days, 5 µg/mouse/day) or saline (control). Supernatants from ConA activated cells were subjected to ELISA analysis of TNF-α and IFN-γ. The amounts of secreted TNF-α and IFN-γ were decreased following administration of heparanase (□) as compared to saline (▪). **C. In vitro.** C57BL/6 derived spleen lymphocytes were harvested and co-activated with IL-2 (24 h, 37°C, RPMI + 10% FCS) in the presence of 65 kDa latent heparanase (30 µg/ml) (□) or saline (▪). Aliquots of the culture medium were subjected to ELISA analysis as above. Each bar represents mean±SD from triplicate wells. All experiments were performed at least three times; variations between different experiments did not exceed ±15%. The amounts of the secreted Th2-type cytokines IL-6 and IL-10 were increased following exposure to heparanase as compared to saline. In contrast, there was a marked decrease in the level of IL-12, representing a Th1 cytokine, in cells that were similarly treated with IL-2 and heparanase for 24 h.

Development of Th1 or Th2 cells from naïve CD4+ T-cells is determined by the cytokine milieu during the initial phase of the immune response. CD4+ T-cells from donor origin with polarization towards the Th1 phenotype exhibit accelerated alloimmune activity against components of their target organs, causing the symptoms observed in GVHD [35]. Increasing the level of cytokines characteristic of Th2 cells and a parallel decrease in the amount of Th1-type cytokines, ameliorate the signs and symptoms of the disease [35]. IL-12 plays a major role in driving differentiation of uncommitted T-cells towards a Th1 phenotype [52]. Conversely, IL-4, IL-6 and IL-10 are produced primarily by Th2 cells [53]. We therefore studied the effect of heparanase on the Th1/Th2 balance in mice. For this purpose C57BL/6 mice were subjected to a daily injection of active (8+50 kDa) heparanase (3 days, 5 µg/mouse/day). Splenocytes were then harvested, activated with ConA (24 h, 37°C, RPMI + 10% FCS) and the levels of secreted IL-4, IL-6, IL-10 and IL-12 were determined by ELISA. The amount of Th2-type cytokines such as IL-4, IL-6 and IL-10, was increased 25.0, 17.2, and 2.2 fold, respectively, following exposure of mice to heparanase (p<0.001, Fig. 4A). In contrast, under the same conditions, there was a marked decrease (8.3 fold, p<0.01) in the level of IL-12 (Fig. 4A). We also studied the effect of heparanase on TNF-α and IFN-γ. TNF-α promotes inflammation and its neutralization suppresses a broad spectrum of inflammatory autoimmune diseases [39], [54]. Similarly, IFN-γ is a potent pro-inflammatory cytokine elevated in both acute and chronic GVHD [55]. The amounts of both TNF-α and IFN-γ secreted by ConA activated splenocytes derived from heparanase treated C57BL/6 mice were 2-fold lower than those secreted by splenocytes derived from untreated mice and subjected to ConA activation (p<0.01, Fig. 4B).

#### Effect of heparanase on cytokine production in vitro

Spleen lymphocytes harvested from C57BL/6 mice were co-activated with IL-2 (24 h, 37°C, RPMI + 10% FCS) in the presence of latent heparanase (30 µg/ml), or saline alone (control group). Aliquots of the culture medium were subjected to ELISA analysis of IL-6, IL-10, and IL-12 levels. The amounts of secreted IL-6 and IL-10, representing Th2-type cytokines, were increased by 12.8 and 14.5-fold, respectively (p<0.001, Fig. 4C), following exposure to heparanase *in vitro*, as opposed to a marked decrease (5.8 fold) in the level of IL-12 (p<0.01, Fig. 4C). Altogether, these results indicate that both active and latent heparanase exert a direct effect on splenocytes, modulating their Th-1/Th2 balance.

## Discussion

Allogeneic SCT is increasingly used for the treatment of a growing number of malignant and non-malignant disorders. However, GVHD remains a major obstacle [30]. Heparanase, the predominant enzyme degrading heparan sulfate, plays a significant role in inflammation, exerting both enzymatic and non-enzymatic activities [9], [10], [15]. Yet, the involvement of heparanase in GVHD has not been evaluated. Our results indicate an important role of heparanase in facilitating engraftment and suppressing GVHD post SCT, critical to the success of hematopietic transplantation.

We have demonstrated that heparanase facilitates engraftment as indicated by a higher WBC counts in the peripheral blood of the heparanase treated mice, 2 and 3 weeks post transplantation, as compared to control mice. Degradation of various components of the subendothelial ECM is mandatory for extravasation and transmigration of circulating hematopoietic stem and progenitor cells. Cleavage of HS disintegrates the supramolecular structure of the subendothelial basement membrane, thereby facilitating trans-endothelial migration of hematopoietic cells [25], essential for engraftment. Indeed, Spiegel et al [31] have recently demonstrated a marked increase in the number of hematopoietic stem cells in the BM of heparanase over-expressing transgenic (*Hpa-tg*) mice and that a limited dose of WBC from the BM of these mice was sufficient to rescue lethality irradiated recipient mice [31]. This result further substantiates the relevance of heparanase for hematopoietic engraftment.

Applying mouse transplantation models, we have demonstrated up to 100% survival of both heparanase treated and heparanase over-expressing mice, likely attributed not just to the above described improved engraftment, but primarily to a marked suppression of GVHD.

Heparanase was previously demonstrated to facilitate lymphocyte invasion through tissue barriers [24], [25]. The enzyme has also been shown to release various growth factors, cytokines and chemokines sequestered by HS in the ECM, basement membrane and cell surfaces [16]. The released factors mediate processes such as angiogenesis and cell proliferation that often accompany the inflammatory response, including GVHD [16], [25]. Recently we have demonstrated an association between heparanase gene SNPs and GVHD in patients undergoing allogeneic stem cell transplantation [56]. Discrepancy in heparanase gene SNPs combinations between recipients and donors was found to be a risk factor for developing acute GVHD [56]. It was therefore expected that administration of heparanase to mice in the context of allogeneic SCT will enhance the allo-inflammatory process through increased recruitment of T cells to the affected tissue, accelerating GVHD. In contrast, we observed that administration of heparanase to the donor and recipient mice resulted in a significant decrease in the clinical parameters of GVHD and prolonged survival compared to control saline-treated mice. These results were further substantiated by using transgenic (*hpa-tg*) mice over-expressing heparanase, as recipients of the allogeneic graft. The survival of these mice was 100%, as opposed to 0–25% survival (depending on the dose of transplanted stem cells) in the control mice. Notably, it appears that exogenously added recombinant heparanase is less effective than the endogenous enzyme (*Hpa-tg* mice) in its ability to suppress GVHD, most likely due to pharmacokinetic considerations.

While heparanase enzymatic activity is traditionally involved in promoting lymphocyte cell migration and invasion, the currently observed anti-GVHD effect of heparanase may suggest that a non catalytic mechanism is responsible. In fact, we have identified several non-catalytic activities of heparanase (i.e., cell adhesion, gene transcription, signal transduction) [9], [51], [57] thought to be mediated by yet unidentified cell surface receptors [57]. More recently, we have demonstrated stimulated production and secretion of cytokines, upon incubation of monocytes with inactive heparanase (Blich et al., in preparation), further illustrating the relevance of heparanase non-enzymatic functions to inflammatory processes and possibly GVHD. Of specific relevance is the up-regulation of both VEGF-A [58] and VEGF-C [59] in response to over-expression or exogenous addition of either active or inactive (double mutated) heparanase. Indeed, inverse correlation between VEGF levels and the severity of GVHD were reported (patients with severe GVHD had significantly lower VEGF levels than those with mild or no GVHD, indicating that higher VEGF levels post-transplantation may protect against the development of severe GVHD [60]. To better elucidate the mode of action of heparanase in the GVHD mouse model, we investigated its effect on T cells *in vitro*. Applying the ConA and MLC lymphocyte activation assays, we have demonstrated a direct inhibitory effect of heparanase, either the active (8 + 50 kDa) or latent (65 kDa) forms, on lymphocyte cell activation and proliferation. Moreover, lymphocyte activation by ConA was inhibited also following co-administration of heparanase and a potent inhibitor (non-anticoagulant glycol split heparin) of its enzymatic activity [15], [45], [46], and, even more so, by an inactive enzyme in which the active site proton donor (Glu-225) and nucleophil (Glu-343) [43] were replaced by inert residues. Altogether, these results support the notion that heparanase enzymatic activity is not required to ameliorate the clinical signs of GVHD.

Historically, acute GVHD has been considered a primarily Th1/Tc1-type process based on the predominant of cytotoxic T-cell mediated pathology and increased production of Th1 type cytokines, including IL-12 and IFNγ, while cytokines that polarize donor T-cells to Th2 (e.g., IL-4, IL-10) can reduce acute GVHD [33], [34], [35]. Th2-shifting of lymphocytes is known to be associated with suppression of disease signs in the GVHD model [28], [33], [35], [50]. Notably, we have demonstrated that the stimulated lymphocytes were shifted towards the Th2 phenotype, characterized by increased production of IL-4, IL-6 and IL-10, and a marked decrease in IL-12, TNF-α and IFN-γ secretion by the activated cells. A similar shift was noted in splenocytes derived from C57BL/6 mice that were treated with heparanase. The cytokine release assays *in vitro* were performed with un-separated lymphocytes, resembling the situation *in vivo*. These results support a role for heparanase in determining the polarity status of lymphocytes in a way that suppresses their ability to promote the allo-inflammation process of GVHD. It should be noted, however, that recently a third T cell subset, Th17, has been recognized and suggested to contribute to development of GVHD [61]. Thus, the Th1/Th2 theory may be somewhat oversimplified. The mechanism by which heparanase induces a shift from Th1 toward a Th2 phenotype is not clear. We have previously demonstrated that heparanase enzymatic activity is involved in shedding of syndecan-1(sdc-1) [62]. Sdc-1 modulates inflammatory responses possibly via shifting the Th1/Th2 balance towards a Th2 response, illustrated by decreased Th1 and higher Th2 cytokine/chemokine expression in sdc-1 null mice [63], [64]. Removal of sdc-1 by heparanase may thus provide a mechanism for its anti-GVHD protective effect. Alternatively, the observed anti-GVHD effect of heparanase may be mediated by its non-enzymatic functions, as mentioned above, possibly through binding to and activation of a putative cell surface receptor [57]. Apart of HSPGs, several cell surface proteins have been shown to bind heparanase and mediate its uptake. These include mannose 6-phosphate receptor (MPR) and low density lipoprotein receptor-related protein (LRP) [65], [66] which potentially can mediate heparanase signaling and non-enzymatic effects. The existence of cell surface heparanase receptor(s) is supported by binding experiment, reinforcing the notion that while HSPGs serve as low affinity, high abundant binding sites, heparanase also associates with high affinity, low abundant cell surface receptor(s) [67]. A first indication for the protein nature of this receptor and its molecular weight emerged from cross-linking experiments applying several cell types and revealing two distinct complexes representing 110 and 150 kDa proteins associated with the heparanae C-terminus domain (C-domain) [57]. Clearly, identification and characterization of a cell surface receptor for heparanase constitutes a most relevant challenge for progress in the field. Regardless of its mode of action, the observed effect of heparanase on engraftment, its ability to ameliorate GVHD and improve survival post SCT in mice may be of clinical significance serving as a new strategy, improving the outcome of SCT.
